# *Trypanosoma cruzi* Parasite Load Modulates the Circadian Activity Pattern of *Triatoma infestans*

**DOI:** 10.3390/insects13010076

**Published:** 2022-01-10

**Authors:** Francisco Chacón, Catalina Muñoz-San Martín, Antonella Bacigalupo, Bárbara Álvarez-Duhart, Rigoberto Solís, Pedro E. Cattan

**Affiliations:** 1 Laboratorio de Ecología, Facultad de Ciencias Veterinarias y Pecuarias, Universidad de Chile, Santiago 8820808, Chile; fachacon@veterinaria.uchile.cl (F.C.); catasanmartin@ug.uchile.cl (C.M.-S.M.); abacigalupo@uchile.cl (A.B.); barbara.biri@gmail.com (B.Á.-D.); 2 Programa de Doctorado en Ciencias Silvoagropecuarias y Veterinarias, Campus Sur, Universidad de Chile, Santiago 8150215, Chile; 3 Núcleo de Investigaciones Aplicadas en Ciencias Veterinarias y Agronómicas, Campus Providencia, Universidad de las Américas, Santiago 7500975, Chile; 4 Institute of Biodiversity, Animal Health and Comparative Medicine, University of Glasgow, Glasgow G12 8QQ, UK

**Keywords:** Triatominae, behavior, circadian rhythm, host–parasite interactions, insect vector, locomotion, *Triatoma infestans*, *Trypanosoma cruzi*, parasite load, Chagas disease

## Abstract

**Simple Summary:**

We studied the locomotor activity of one of the kissing bug species that transmit the Chagas disease-causing parasite in humans, which usually bites during the night. To date, no other reports researching its behavior take into account the amount of parasites inside the kissing bug; however, some studies have demonstrated that the presence of parasites modifies the activity of some kissing bug species. We recorded their movements in light and dark conditions after part of the insects fed on mammals that had the parasite and others fed on those that did not have the parasite. Later, their amounts of parasites were quantified. We found that, compared with insects with no parasites, kissing bugs with higher parasite amounts increase the number of times they move and the distance they travel, especially during daylight hours. This could imply that the insect increases its time searching for a food source when it is infected with a higher number of parasites, and this could increase the risk of transmission of the parasite to people by the kissing bug.

**Abstract:**

American trypanosomiasis is a disease caused by the flagellate protozoan *Trypanosoma cruzi*, which is transmitted mainly in endemic areas by blood-sucking triatomine vectors. *Triatoma infestans* is the most important vector in the southern cone of South America, exhibiting a nocturnal host-seeking behavior. It has been previously documented that the parasite produces changes in some triatomine species, but this is the first time that the behavior of a vector has been evaluated in relation to its parasite load. After comparing the movement events and distance traveled of infected and non-infected *T. infestans*, we evaluated the change produced by different *T. cruzi* parasite loads on its circadian locomotor activity. We observed differences between infected and non-infected triatomines, and a significant relation between the parasite load and the increase in locomotor activity of *T. infestans*, which was accentuated during the photophase. This could have direct implications on the transmission of *T. cruzi*, as the increased movement and distance traveled could enhance the contact of the vector with the host, while increasing the predation risk for the vector, which could both constitute a risk for vectorial and oral transmission to mammals.

## 1. Introduction

American trypanosomiasis (Chagas disease) is a zoonotic parasitic disease endemic to the American continent. Its etiological agent is *Trypanosoma cruzi* (Chagas, 1909) (Trypanosomatida: Trypanosomatidae), which is mainly transmitted through the dejections of triatomine bugs [1]. Triatomines’ nymphs and adults are strict blood-sucking insects that feed on vertebrate species available in their habitat [2]. *Triatoma infestans* (Klug, 1834) (Hemiptera: Reduviidae) is the main vector of this disease in the southern cone of South America, due to its high adaptation to human dwellings [3,4]. However, sylvatic foci have been reported in several countries [5,6,7,8,9], hindering the hope of eradicating this vector [4].

Behavioral changes have been described in host–parasite systems, and a parasite may alter more than one distinctive trait of its host [10]. The evidence indicates that triatomines could present behavioral changes associated with their *T. cruzi*-infection status, with reports on the modification of their biting frequency and locomotor behavior in *Mepraia spinolai* (Porter, 1934), *Rhodnius prolixus* (Stal, 1859), *Triatoma pallidipennis* (Stal, 1945), and *Triatoma longipennis* (Usinger, 1939) (Hemiptera: Reduviidae) [11,12,13,14]. Infected *T. longipennis* and *T. pallidipennis* react more quickly to human odor than non-infected nymphs [13]. Infected *T. infestans* nymphs defecate in less time and deposit greater amounts of dejections than non-infected nymphs [15]. Infected *M. spinolai* nymphs orient towards their host twice as fast, their number of bites is doubled, they defecate earlier than non-infected nymphs [11], and they increase their movement events in scotophase (darkness phase), without showing significant differences during photophase (light phase) [14]. In contrast, *T. cruzi*-infected *R. prolixus* decrease their movement events by about 20% compared to non-infected ones, mainly at the beginning of the scotophase [12]. Some of these behavioral changes could promote vector competence, which encompasses the ability to acquire, maintain, and transmit a pathogen [16]; in triatomines, it associates with host-seeking, feeding, and defecation behavior [15], among other traits.

Most triatomine species are nocturnal insects, including *T. infestans* [17], and they normally present a bimodal pattern of activity [18], with peaks that occur when the intensity of light decreases and during the first hours of the day, returning to their shelters before dawn [18,19]. Any change in the behavior of triatomines at the level of their circadian rhythm; i.e., the daily rhythmic activity cycle that is related to light and darkness, would modify important basic biological processes, such as dispersal and search for hosts [20,21,22], so it would translate into relevant changes in the interaction between the triatomine, the parasite, and the host [12,23].

To our knowledge, the relation between the *T. cruzi* parasite load on the vector and the behavior of *T. infestans* has not been reported previously in the literature. In this study, we evaluate the changes in the circadian locomotor activity of the nocturnal vector *T. infestans*, while infected and non-infected with *T. cruzi,* and we evaluate the relation of the behavior with different ranges of parasite load.

## 2. Materials and Methods

*Triatoma infestans* individuals were obtained from a colony maintained in the Laboratorio de Ecología, Facultad de Ciencias Veterinarias y Pecuarias, Universidad de Chile, where they were kept under controlled environmental conditions of 25 °C with 70% relative humidity, in a light:darkness cycle (L:D, hereafter) of 12 h each, and fed monthly with chicken blood.

Nymphal stages were determined following Brewer et al. (1981) [24]. Considering that only in the adult stage is it possible to differentiate the sex by morphology in this species, the nymphal stages were studied without differentiation by sex.

The control group included 26 nymphs of stage III that were fed on non-infected *M. musculus* (Linnaeus, 1758) (Rodentia: Muridae). Thirty-nine nymphs of stage III and 17 of stage IV, were fed on naturally *T. cruzi*-infected *Octodon degus* (Molina, 1782) (Rodentia: Octodontidae) that were previously captured from the field, and 26 nymphs of stage V fed on experimentally infected *M.* *musculus*. Triatomines were fed on anesthetized hosts. Briefly, *O. degus* were induced with isoflurane and later injected with ketamine/xylazine; *M. musculus* were directly anesthetized with ketamine/xylazine [25,26].

The *M. musculus* infection was carried out with 1000 parasites of the Dm28c strain of *T. cruzi* in 100 microliters of rpmi 1640 medium (Biological Industries™) inoculated once per animal by the intraperitoneal route. The effective infection of the mice was subsequently evaluated by light microscopy of the blood samples after 7 days, and triatomines were fed on them when they achieved 80,000–500,000 parasites/mL.

Before triatomine feeding, both rodent species were kept in mesh-covered acrylic cages, with wood chips as bedding, and with water and food ad libitum. After triatomine feeding, the *M. musculus* were euthanized using an excess of anesthetics and extraction of cardiac blood.

After feeding, each triatomine was maintained individually in a plastic flask with a perforated screw cap, provided with folded paper as a refuge, and all flasks were placed inside a climatic chamber with controlled environmental conditions consisting of an L:D cycle of 12:12 h, temperature of 27 ± 1 °C, and 70 ± 10% relative humidity.

### 2.1. Circadian Locomotor Activity

The triatomine behavioral experiments began approximately 45 days after feeding on the infected or non-infected hosts, and were performed in a circular experimental glass arena of 20 cm in diameter, inside an experimental chamber with controlled light, humidity, and temperature.

We recorded the number of movement events, defined as a change in position comprising not less than the body length of the individual. We also recorded the total distance traveled in the experimental arena. Both measurements were recorded during the 12 h of the dark phase (scotophase) and the 12 h of the light phase (photophase). A 40 w bulb was available as a light source for the photophase, which produced a maximum intensity of 65 lux. Infrared (IR) light was used during the scotophase, creating functional conditions of darkness [27].

The activity was recorded continuously for 24 h with a sampling rate of 7.4 frames per second (FPS, hereafter) and the recordings were analyzed by the Noldus Ethovision XT14 Software^®^.

### 2.2. Detection and Quantification Trypanosoma cruzi DNA

The insects were euthanized by freezing overnight 5 ± 2 days post behavioral experiment. DNA was extracted from the abdomen of each insect, cut using a new scalpel per individual. The abdomen was homogenized using a Lysing Matrix I (MP Biomedicals™) in a FastPrep24 instrument (MP Biomedicals™). After, the DNA was extracted using a commercial kit (innuPREP DNA Mini Kit, Analytik Jena™) according to the manufacturer’s recommendations, following the protocol for DNA extraction from tissue samples and modifying the lysis time to 60 min, vortexing every 10 min. The extracted DNA was stored at −20 °C until use.

To detect and quantify *T. cruzi* DNA in *T. infestans,* real-time PCR assays were performed targeting DNA from a conserved satellite region. The reaction mix contained 2 μL of the sample, 4 μL of 5X HOT FIREPol^®^ EvaGreen^®^ qPCR Mix Plus (Solis BioDyne), 0.3 µM of each of the primers Cruzi 1 (5′-ASTCGGCTGATCGTTTTCGA-3′) and Cruzi 2 (5′-AATTCCTCCAAGCAGCGGATA-3′) [28], and nuclease-free water, obtaining a final reaction volume of 20 μL.

The standard curve for the absolute quantification of *T. cruzi* was composed of 8 points ranging from 10^6^ to 10^−1^ parasite equivalents/mL in 1:10 serial dilutions of an equal mixture of the genomic DNA of the *T. cruzi* strains DM28c (TcI) and Y (TcII), due to the variability in copy number of the satellite region of *T. cruzi* according to its DTU [29,30,31]. The number of parasites was estimated considering the amount of DNA equivalent to 1,000,000 parasites/mL, given that 1 parasite contains approximately 200 fg of DNA [29,32].

To quantify *T. infestans* DNA as the endogenous control, we amplified a conserved region of the 18S subunit of the ribosomal DNA in duplicate, using the reaction mixture described above but with the primers 18S For (5′-TCCTTCGTGCTAGGAATTGG-3′) and 18S Rev (5′-GTACAAAGGGCAGGGACGTA-3′) [32], for a final reaction volume of 20 µL. We generated a 4-point standard curve, ranging from 0.86 µg/mL to 0.86 × 10^−3^ of *T. infestans* 18S.

Both reactions were prepared in a laminar flow hood and direct light on the workspace was avoided. The amplification was run in a Rotor-Gene^®^ Q thermal cycler (2plex HRM Platform). The amplification program consisted of an incubation at 95 °C for 15 min and 40 cycles consisting of: 95 °C for 15 s; 65 °C for 20 s; and 72 °C for 20 s. The recording of the emitted fluorescence was carried out at 72 °C at the end of each cycle. The following were included in each run: nuclease-free water instead of a sample as a no-template control; a control with DNA extracted from infection-free *T. infestans* that corresponded to a point of the 18S subunit standard curve; and a control that consisted of one point of the quantification curve of *T. cruzi* DNA, with all samples in duplicate and standard curves in triplicate.

For the normalization of the data, the quantification of *T. cruzi* satellite DNA (par-eq/mL) was divided by the quantification of 18S subunit rDNA (µg/mL) from each individual and multiplied by 1000, obtaining the parasite load as parasite-equivalents/mg of *T. infestans* DNA (par-eq/mg, hereafter) of each triatomine, and allowing the parasite load to be compared among all triatomines, regardless of their weight or size.

### 2.3. Statistical Analyses

We tested the data for normality using the Kolmogorov–Smirnov test and for homogeneity of variance using the Levene test. Differences between the infected and non-infected groups were assessed using the Mann–Whitney U test, and we used the Kruskal–Wallis test for testing differences among nymphal stages. We evaluated the correlation between the parasite load and each behavioral variable with the Spearman test. To analyze the pattern of circadian activity, we performed a Rao spacing test to determine if the data were randomly distributed [33]. In all of these tests, we considered a 95% confidence interval (α = 0.05). Statistical tests were performed with R 4.1.2 (packages: circular, CircStats, Directional and ggplot2), VisualParadigm, and IBM SPSS Statistics v26.

## 3. Results

### 3.1. Triatomine Circadian Locomotor Activity

The results of the Kolmogorov–Smirnov normality test for the movement events in the scotophase, the movement events in the photophase, the distance traveled in the scotophase, and the distance traveled in the photophase, indicated that these variables were not normally distributed (Table S1). The detailed results of the circadian locomotor activity for each individual are available in the Supplementary Materials (Table S5).

### 3.2. Detection and Quantification of Trypanosoma cruzi DNA

From the 82 triatomines fed on infected hosts, 26 were negative for *T. cruzi*, all fed on chronically infected *O. degus*; therefore, we considered them as part of the non-infected group for future analyses. This gives a rate of 54% of successful triatomine infections while feeding on this naturally infected host species. The *T. cruzi* parasite loads of the 56 positive insects ranged from 0.8 to 4,867,678 par-eq/mg, with a median of 4046.9 par-eq/mg (Table 1), without significant differences on the parasite loads of positive triatomines among feeding sources: chronically infected *O. degus* and acutely infected *M. musculus* (Kruskal–Wallis test *p* = 0.73; Table S3) and without significant differences on the parasite loads of the infected nymphs among stages (Kruskal–Wallis test *p* = 0.266; Table S4). The detailed results for the real-time PCR runs and parasite loads are available in the Supplementary Materials (Table S5).

### 3.3. Triatomine Circadian Locomotor Activity and Parasite Load

**Figure 1:**
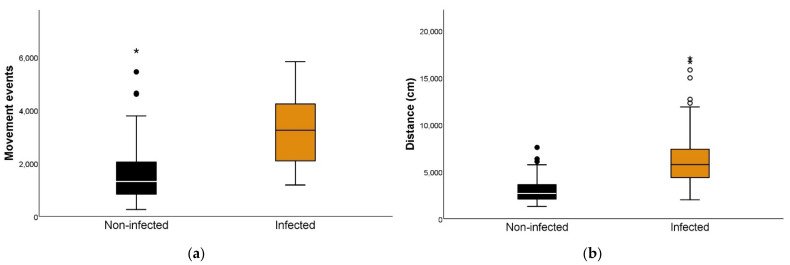
Locomotor activity according to the *Trypanosoma cruzi* infection status of *Triatoma infestans*. (**a**) Number of movement events during 24 h of *T. cruzi*-infected and non-infected *T. infestans* (Mann–Whitney U test *p* < 0.001). A movement event is defined as a change in position comprising not less than the body length of the individual. (**b**) Distance traveled in centimeters during 24 h in *T. cruzi*-infected and non-infected *T. infestans* (Mann–Whitney U test *p* < 0.001). (•) represent outliers: a data point is considered an outlier if it is more than 1.5 times the inter-quartile range above the third quartile or below the first quartile. (*,**) represent extreme outliers: a data point is considered an extreme outlier if it is more than 3 times the interquartile range above the third quartile or below the first quartile.

First, we separated the triatomines into an infected group and a non-infected group. When comparing each activity variable for both groups, there were significant differences, with infected individuals demonstrating higher figures for movement events and distance traveled in scotophase and especially during photophase (Figure 1, Table 2, and Table S2).

**Figure 2:**
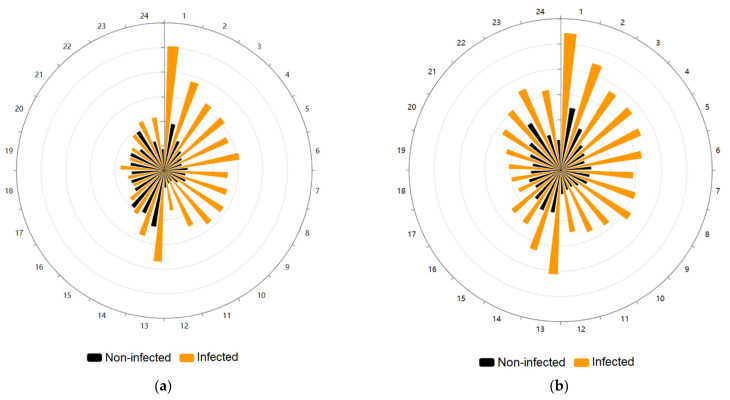
Circular plots showing the locomotor activity per hour of *Triatoma infestans*. The photophase spans from the first to 12 h, and the scotophase from 13 to 24 h. (**a**) Movement events per hour of *T. infestans*. The radius is divided into six equal parts, each of 3000 units (movement events). Orange-colored bars show the sum of movement events of the *Trypanosoma* *cruzi*-infected individuals and black-colored bars show the non-infected specimens. (**b**) Distance traveled per hour by *T.* *infestans*. The radius is divided into six equal parts, each of 5000 units (centimeters). Orange-colored bars show the sum of the distance traveled in centimeters of *T. cruzi*-infected individuals and black-colored bars show the non-infected specimens.

When we plotted the sum of movement events of infected and non-infected specimens, we clearly observe the difference during photophase between both groups, with infected individuals moving at least three times more frequently than the non-infected ones, while during scotophase this difference is reduced, occurring mainly at the beginning and at the end of this period (Figure 2a). The pattern of movement events was not randomly distributed for both the infected (Rao spacing test *p* < 0.001) and the non-infected group (Rao spacing test *p* < 0.001).

The plot of the sum of the distance traveled by both groups demonstrates the remarkable difference between them, with infected individuals at least doubling the distance traveled by non-infected specimens both at photophase and scotophase (Figure 2b). The pattern of the distance traveled was not randomly distributed again for the infected (Rao spacing test *p* < 0.001) and non-infected *T. infestans* (Rao spacing test *p* < 0.001).

### 3.4. Parasite Load Effect on the Movement Events of Triatoma infestans

**Figure 3:**
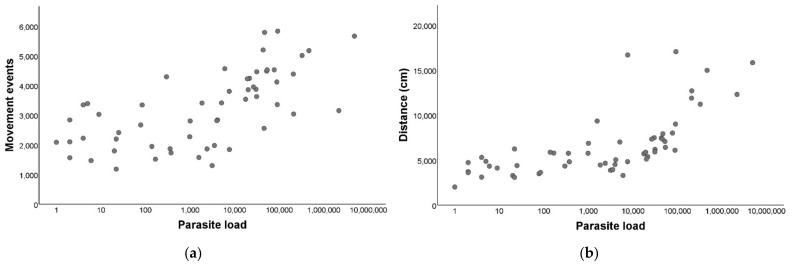
Locomotor activity in relation to *Trypanosoma cruzi* parasite load of *Triatoma infestans*. (**a**) Parasite load (LOG_10_) in relation to the number of movement events during 24 h. (**b**) Parasite load (LOG_10_) in relation to the distance traveled in centimeters during 24 h.

After establishing that there were differences between the *T. cruzi*-infected and non-infected triatomines, we performed a correlation to evaluate if there was a relation between the parasite loads and the movement events within the group of infected *T. infestans*, and we observed a marked positive and significant relation (r_s_ = 0.69; *p* < 0.001; Figure 3a).

### 3.5. Parasite Load Effect on the Distance Traveled by Triatoma infestans

We performed a correlation to evaluate if there was a relation between the parasite load and the distance traveled and we detected a significant positive relation (r_s_ = 0.79; *p*< 0.001; Figure 3b).

## 4. Discussion

To our knowledge, this is the first study to address the effect of the parasite load on the behavioral response of triatomines, quantifying *T. cruzi* and evaluating the circadian locomotor behavior of *T. infestans*. To accomplish this, we measured the movement events and the distance traveled of triatomines; each specimen was evaluated for 12 h in scotophase and 12 h in photophase.

The parasite loads obtained from the 56 positive individuals demonstrated a fairly wide range (from 0.8 to 4,867,678 par-eq/mg) and were not normally distributed. This was also detected in previous studies reporting the *T. cruzi* parasite load in *T. infestans* dejections [34,35] and in the intestinal content of *R. prolixus* [36]. This wide range in vectors has been related to the acute and chronic infection in host mammals, due to the number of parasites ingested by the triatomines [37,38,39,40]; however, when we compared the parasite load according to the feeding sources, the chronically infected *O. degus* and acutely infected *M. musculus*, we did not detect significant differences. Some of the *T. infestans* feeding on *O. degus* did not acquire the infection; this is related to the infectiousness of chronically infected mammal hosts, namely, low parasitemia [41,42].

We detected significant differences between infected and non-infected triatomines, in movement events and distance traveled. Non-infected triatomines displayed a lower activity in general, being preferably nocturnal; however, *T. cruzi*-infected triatomines show an increase in locomotor activity in both phases, being higher during the photophase. This finding was unexpected, as previous reports demonstrated a reduction in the movement events of *R. prolixus*, another nocturnal triatomine, when infected with *T. cruzi*; however, the same triatomine species presented an increase in its movement events during the photophase when infected with *Trypanosoma rangeli* (Tejera, 1920) (Trypanosomatida: Trypanosomatidae), which has been described as a pathogen for triatomines [12,43]. An increase in movement events was observed as well for infected *M. spinolai* compared to non-infected individuals, but this occurred during the scotophase [14]; however, *M. spinolai* is a diurnal triatomine [44], thus it was proposed that this effect could be associated with an increase in its negative phototactic response when infected by *T. cruzi* [12,17]. *Mepraia spinolai* did not show significant differences on the distance traveled when comparing infected and non-infected individuals [14], but activity increases have been described for infected *M. spinolai* and *T. infestans* in other behavioral parameters when compared with non-infected ones, such as feeding and defecation [11,15]. Both *T. longipennis* and *T. pallidipennis* nymphs infected with *T. cruzi* have shown behavioral changes compared to non-infected specimens that resulted in an increase in movements measured as activity time and earlier detection of human odor [13].

It has been previously documented in other insects and crustaceans that infected individuals may present changes in response to light and/or modify their locomotor activity. The cockroach *Periplaneta americana* (Linnaeus, 1758) (Blattodea: Blattidae), when parasitized by *Moniliformis moniliformis* (Bremser, 1811) (Moniliformida: Moniliformidae), increases its locomotor activity and positive phototaxis [45]. The crustacean *Gammarus insensibilis* (Stock, 1966) (Amphipoda: Gammaridae) when infected with *Microphallus papillorobustus* (Rankin, 1940) (Plagiorchiida: Microphallidae), increases its positive phototaxis, negative geotaxis, and aberrant evasion, in combination with an increase in serotonin synthesis and nitric oxide synthase [46,47]. Serotonin has been related to influence behavior in insects, such as circadian rhythms, responses to visual stimuli, and sleep, among others [48], and the nitric oxide synthase is required to produce nitric oxide, which constitutes a signaling molecule in the nervous system of insects [49]. The diurnal mosquito *Aedes aegypti* (Linnaeus, 1762) (Diptera: Culicidae) has also demonstrated an increase in its locomotor activity when infected by *Wolbachia pipientis* (Hertig and Wolbach, 1924) (Rickettsiales: Anaplasmataceae), and when infected by Dengue virus (Rush, 1779) (Amarillovirales: Flaviviridae) [50,51,52]. Dengue infection in this mosquito increased its activity during the whole 24-h cycle; however, this was more evident during the photophase, and its peak occurred during the lights-on transition [53], similar to the changes detected here with the nocturnal *T. infestans*. Infected *A. aegypti* females increase their locomotor activity mediated by sensitivity to human odor, demonstrating differential transcription according to the levels of Dengue virus load assessed [53]. In another mosquito, *Culex tarsalis* (Linnaeus, 1758) (Diptera: Culicidae), the female reduces its flight activity when infected with Western equine encephalomyelitis virus (Meyer, 1931) (Martellivirales: Togaviridae); in addition, this virus generates a reduction in the longevity of the mosquito, and this is dependent on the viral load [54,55]. These are a few of the scarce examples available that evaluate the parasite load in a vector and its relation with the vector’s behavior.

In summary, infection in vectors has been shown to affect their locomotor activity, but the result of this modulation is not necessarily the same for different species within a certain taxon, nor even for the same species exposed to different parasites, thus experimentation is required to determine if there is an effect, and if this will increase or decrease their activity.

In this study, we observed that there is a significant correlation between the *T. cruzi* parasite load and the increase in movement events, associated mainly with an increased activity during the photophase. We also detected a positive and significant correlation between the distance traveled and the *T. cruzi* parasite load, with an increase in both phases, being much greater during the photophase. It has been previously documented that the interaction caused by *T. cruzi* in triatomines could modify their negative phototactic response [17,56]. However, the effect that *T. cruzi* infection would have on locomotor behavior, probably affecting the level of sensitivity response to changes in light conditions [12,17,56], would depend on the range of parasite load, which could explain our discrepancies in the observed effects compared to previous reports, which did not evaluate the parasite load of their infected specimens [17]. In addition, when the lights are turned on, they generate an increase in locomotion as a consequence of the sudden change in intensity, regardless of having an additional circadian component [12]. This was observed in our study, with peaks both at the beginning of the experiment during the photophase and also at the beginning of the scotophase, when exposed to sudden darkness conditions.

In our study, non-infected *T. infestans* behaved as normally described for nocturnal triatomines, reducing their activity during daylight hours, with increases before dawn and in the first hours after dusk [57]. We observed the opposite in the infected group, with an increase in movement events and distance traveled during the photophase, the period during which the nocturnal *T. infestans* would normally stay in its shelter. This modification may be considered a potentially costly behavioral modulation of the parasite on triatomines [58,59], as an increase in positive phototaxis could lead to greater exposure to predators [12], but it could also increase the vector’s probability of finding hosts [18]. Predation on the vector could turn into a beneficial outcome for the parasite, as oral transmission to mammals is described for *T. cruzi* [60,61].

Considering the hypothesis of sensory modulation by the parasite [12,17], which in this case would be exacerbated particularly during the photophase for infected *T. infestans*, another possibility could be that the negative phototactic response of these insects forced them to actively search for shelter, and this was visualized as an increase in movement events and distance traveled during the light phase. We did not include any structure in the experimental arena that could be used as a cover to avoid light, as losing sight of the individual interfered with the tracking system.

Many variables could be influencing the different findings of this study compared with the literature; among these are the triatomine species evaluated, the *T. cruzi* parasite strains infecting the vectors, different periods of starvation [11,57], and probably, as indicated by our results, different parasite load ranges in the vectors.

Considering that the locomotor activity mediates the interaction between the triatomine vector and the host as food source [12], our results demonstrate behavioral changes in *T. infestans* that could favor the transmission of *T. cruzi*, modifying its vectorial competence [15]. Future studies should examine in depth the effect of the parasite load on other aspects of the behavior of triatomine insects, such as feeding and defecation, which could constitute epidemiologically relevant behavioral changes, and evaluate sexual behavioral changes in adults, which could impact the growth of triatomine populations. Particularly important would be to study their photoreceptors, and to clarify if there is a threshold of behavioral response that depends on the *T. cruzi* parasite load.

## Figures and Tables

**Table 1:** Number of *Triatoma infestans* infected by *Trypanosoma cruzi* per parasite load range.

Parasite-Equivalents/ Mg of *Triatoma infestans* DNA	N of *Triatoma infestans*
<1	1
1–<10	8
10–<100	6
100–<1000	6
1000–<10,000	12
10,000–<100,000	17
100,000–>100,000	6

**Table 2:** Summary values of movement events and distance traveled by non-infected and infected *Triatoma infestans*.

	Infection Status	N	Units	Median	Mean	SE	Min	Max
**Movement events in photophase**	Non-infected	52	Events	589.5	635.88	39.16	168	1585
	Infected	56	Events	1596	1853.69	99.99	846	3753
**Movement events in scotophase**	Non-infected	52	Events	664.5	1059.1	161.13	22	4808
	Infected	56	Events	1328	1314.89	110.69	109	3190
**Movement events total**	Non-infected	52	Events	1293.5	1694.98	182.94	258	6232
	Infected	56	Events	3188	3168.57	167.02	1180	5829
**Distance traveled in photophase**	Non-infected	52	cm	1340.4	1406.37	54.43	743.8	2191.1
	Infected	56	cm	2895.1	3514.61	242.08	1063.4	8735.2
**Distance traveled in scotophase**	Non-infected	52	cm	1501.55	1673.48	166.44	321.6	5603.2
	Infected	56	cm	2221.35	2826.7	293.33	513.9	13,533.4
**Distance traveled total**	Non-infected	52	cm	2672.15	3079.85	190.32	1299.3	7573
	Infected	56	cm	5724.15	6341.31	442.87	2008.9	17,039.8

## Data Availability

Data are contained within the article or Supplementary Materials.

## Supplementary Materials

The following supporting information can be downloaded at: https://www.mdpi.com/article/10.3390/insects13010076/s1, Table S1: Results of the Kolmogorov–Smirnov test for normality and the Levene test for homogeneity of variance, for each of the behavioral variables studied; Table S2: Results of the Mann–Whitney U test comparing the movement events and distance traveled by non-infected (*n* = 52) and infected (*n* = 56) triatomines; Table S3: Results of the Mann–Whitney U test comparing the parasite load of triatomines fed on each rodent species: chronically infected *Octodon degus* and acutely infected *Mus musculus*; Table S4: Results of the Kruskal–Wallis test comparing the parasite load among nymphal stages (III, IV, and V); and Table S5: Compiled database with all individuals tested and their results for each of the variables analyzed.
