# Supplementary material for: Trypanosoma cruzi Parasite Load Modulates the Circadian Activity Pattern of Triatoma infestans
Source: Insects. 2022 Jan 10;13(1):76. doi: 10.3390/insects13010076 (PMC8777832; doi:10.3390/insects13010076)
Supplement: Supplementary file 1 [file insects-13-00076-s001.zip › Supplementary tables.pdf]

Table S1. Results of the Kolmogorov-Smirnov test for normality and the Levene test for homogeneity of variance, for each of the behavioral variables studied, comparing *Trypanosoma cruzi* infected with non-infected *Triatoma infestans*.

	**Kolmogorov-Smirnov**	**Levene test**
Movement events in photophase	p<0.001	p=0.571
Movement events in scotophase	p<0.001	p=0.537
Movement events Total	p=0,001	p=0.235
Distance traveled in photophase	p<0.001	p=0.591
Distance traveled in scotophase	p<0.001	p=0.091
Distance traveled Total	p<0.001	p=0.374

Table S2. Results of the Mann-Whitney U test comparing the movement events and distance traveled, by non-infected (n= 52) and *Trypanosoma cruzi* infected (n= 56) triatomines.

	**Mann-Whitney U test**
Movement events in photophase	p<0.001
Movement events in scotophase	p=0,014
Movement events Total	p<0.001
Distance traveled in photophase	p<0.001
Distance traveled in scotophase	p<0.001
Distance traveled Total	p<0.001

Table S3. Results of the Mann-Whitney U test comparing the parasite loads of triatomines fed on each rodent species: chronically infected *Octodon degus* and acutely infected *Mus musculus*.

	**N°**	**Mann-Whitney U test**
Infected triatomines	56	p=0.730
Movement events in scotophase	82	p<0.001

Table S4. Results of the Kruskal-Wallis test comparing the parasite loads between nymphal stages (III, IV and V).

	Kruskal-Wallis test		
	**N°**	**Statistic**	**Sig**
Infected triatomines	56	2.651	p=0.266
Movement events in scotophase	82	14.85	p=0.001
